# Effects of aircraft operating fluids and environmental thermal fatigue on fly ash and steel slag based cementitious composites

**DOI:** 10.1038/s41598-024-63558-y

**Published:** 2024-06-03

**Authors:** Aniruddha Tangirala, S. Rawat, Mukund Lahoti

**Affiliations:** 1 Department of Civil Engineering, Birla Institute of Technology and Science, Pilani, Pilani, 333031 India; 2 School of Engineering, Design and Built Environment, Western Sydney University, Penrith, NSW 2751 Australia

**Keywords:** Environmental thermal fatigue, Airfield pavement, High-volume fly ash, Steel slag fine aggregates, Aircraft fluids, Aerospace engineering, Engineering, Civil engineering

## Abstract

This paper investigates the performance of concrete incorporating high-volume fly ash (HVFA) and steel slag aggregates against the detrimental effects of combined cycles of environmental thermal fatigue and exposure to leaked aircraft fluids. A total of 128 cubes and 90 prisms were cast for five mixes and exposed to 60, 120, 180, 240 and 300 combined cycles. The results demonstrate the positive effect of utilization of HVFA which reduces the total amount of portlandite available in the system. The SS aggregates demonstrate a strong interlocking with the surrounding matrix and supply the necessary portlandite for continued pozzolanic reaction. However, their reaction with aircraft fluids causes significant degradation to flexural strength initially, which is redeemed by pozzolanic reaction at a later stage. Hybrid basalt and polypropylene fibres were successful in enhancing the flexural strength and reducing the cracking. The mercury intrusion porosimetry revealed a reduction in pore volume because of HVFA. Scanning electron microscopy and differential scanning calorimetry were also employed to uncover the underlying mechanisms of damage and assess the performance of the cementitious composite.

## Introduction

Environmental thermal fatigue (ETF) of concrete refers to the deterioration that occurs in concrete due to a repeated variation in temperature over an extended duration^1^. Typically, the temperature of concrete surfaces during daytime can increase to 60–70 °C even when the ambient atmospheric temperatures is around 30 °C^2^. Further, the concrete temperatures notice a significant change in night or during rainy seasons causing a thermal shock. Over time, the differential thermal expansion and contraction cause microcracks to form and propagate within concrete, leading to a reduction in its durability. This problem is serious in case of the airfield pavements which have large surface areas and cost significantly higher than ordinary pavements. These pavements may require 74 M to 370 million US dollars for their construction and are designed to serve for 20–40 years^3,4^. The closure of a runway at civilian airports results in financial losses, and the risks are significantly higher for strategic military airfield pavements.

Generally, airfield pavements experience fatigue load, impact load, as well as shrinkage in addition to severe weather conditions. Furthermore, they are exposed to hydraulic oil, lubricating fluid, and air traffic fuel leaked from the aircrafts that cause saponification, scaling, chipping, and strength loss of concrete^5–7^. The aircraft fluids consist of esters of fatty acid, carboxylic acid, phosphate esters etc. Saponification occurs when carboxylic acid from the aircraft fluids reacts with the portlandite in the cementitious system to form calcium carboxylate salts (soap) and water at higher temperatures. Formation of carboxylate salts is also possible with the reaction between calcium carbonate in concrete and esters of fatty acids. Furthermore, calcium phosphate salts are produced by the reaction of phosphoric acid (originating from phosphate esters at high temperatures) with portlandite. The penetration of these deleterious fluids through the cracking caused by ETF can potentially increase the rate of degradation and significantly reduce the service life of pavement.

Also, cracks, spalls, and loose fragments from concrete pavement can create debris on runways, taxiways, and aprons which can be called as foreign object debris (FOD) on airfield pavements. They represent a critical concern for aviation safety, potentially causing extensive damage to aircraft and even leading to accidents. Also, the considerable rise in air traffic coupled with ongoing enhancements in payload capacity has led to a manifold increase in the maintenance demands for airfield pavements^8^. Therefore, material selection for construction of airfield pavements must take into account thermal and chemical resistance to extend its service life.

High-volume fly ash (HVFA) based concrete may be suitable in this scenario as it has lower interconnectivity of pores due to the conversion of portlandite to secondary CSH^9–11^ which decreases the ingress of water and other deleterious fluids. The properties of HVFA mixes could be enhanced by blending them with silica fume (SF). This blending enhances abrasion resistance, a property closely linked to the compressive strength^9^, thereby fulfilling a crucial requirement for pavement construction. Recently, newly developed fibre reinforced cementitious composites (FRCC) incorporating HVFA exhibited outstanding thermal resistance at a very high temperature range up to 600 °C^12^. This enhancement in thermal performance was attributed mainly to the incorporation of HVFA, establishing it as a thermal performance enhancer (TPE). Furthermore, utilizing fly ash (FA) in concrete offers multiple benefits, including resource conservation, enhanced rheological properties and lower heat of hydration in concrete and subsequent reduction in concrete cracking^13–17^. Consequently, this matrix formulation may possibly be suitable against ETF to attain high residual strengths.

Further improvement in residual performance of cementitious composites is possible through proper choice of fine aggregates. Steel slag (SS) aggregates which are chemically similar to cement, can help prevent uneven expansion in cement-based materials, consequently improving its residual performance when exposed to higher temperatures^18^. Also, these fine aggregates were previously found to form a very strong bond with the surrounding matrix because of the partial hydration reaction on the surface of the SS particles^19,20^. Therefore, a reduction in the ingress of deleterious fluids can be expected. Furthermore, incorporation of waste generated from steel industry as a replacement of river sand improves the sustainability of the cement composite.

In addition, attention must also be directed towards challenges associated with shrinkage in case of frequent thermal cycles. Incorporation of basalt fibres (BF), the twenty-first century green material, have been found to eliminate the possibility of drying shrinkage^21–23^. Also, BF enhance the tensile and flexural capacities of the cementitious composites that can reduce and delay the cracking caused by shrinkage, thermal variations, and chemical attacks. Polypropylene (PP) fibres have also previously been found beneficial in reducing the shrinkage, thermal cracks, and improving durability^24,25^. Therefore, the hybridization of PP fibres with BF can offer desirable characteristics to cementitious composites while ensuring sustainability.

Despite the continuing advancement in the field of cementitious composites, very few studies have addressed the impact of ETF. Moreover, the combined effects of ETF alongside aircraft fluids on airfield pavements remain unexplored in the scientific literature. Therefore, this study presents the first systematic investigation of the combined influence of ETF and aircraft fluids for pavement materials. From material selection perspective, TPEs in the form of HVFA, sustainable SS aggregates along with BF-PP fibres have been considered to restrict the damage. This has been analysed using scanning electron microscopy (SEM), differential scanning calorimetry (DSC) and mercury intrusion porosimetry (MIP) to provide deeper insights into the underlying mechanisms. This study is novel from an all-round perspective in terms of problem identification and deliverance of a suitable material which can be beneficial for researchers and general practitioners alike.

## Materials and methods

### Materials

The study utilizes ordinary Portland cement (SiO_2_ = 17.55%, Al_2_O_3_ = 3.56%, CaO = 67.98%, Fe_2_O_3_ = 4.98%), class-F FA (SiO_2_ = 59.34%, Al_2_O_3_ = 28.94%, CaO = 1.28%, Fe_2_O_3_ = 5.95%), and SF (SiO_2_ = 95.11%) as binders. Fine aggregates in the form of river sand and SS (34.58% SiO_2_ and 47.03% CaO) passing through 300 µm sieve were used. Polycarboxylate-ether based superplasticizer was used to improve and adjust the flowability of the mixes. 12- and 18-mm long BF and 12-mm long PP fibres were also used.

In addition to the above, the commonly used aircraft fluids in the form of Aeroshell fluid 31 (hydraulic fluid), Aeroshell Turbine Engine oil 500 (lubricating fluid), and Air traffic fuel were used to investigate their impact. These were purchased from Fledge Aero Private Ltd., Delhi.

### Sample preparation and mix proportions

**Figure 1:**
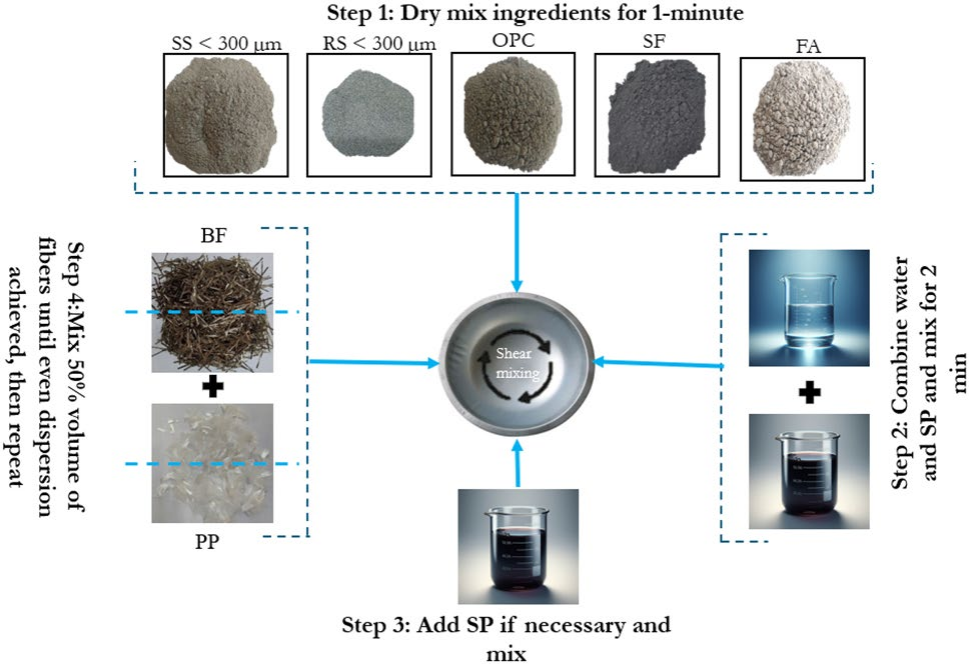
Method of mixing adopted in the study.

Table 1 provides information on the mixes designed for the airfield pavement. These mixes take into consideration the thermal performance, mechanical performance, durability, and sustainability via variations in fibre length and content, and SS fine aggregate content. The mixing method has further been demonstrated in Fig. 1. The process involves dry mixing of the powdered material which included binder (OPC, FA, SF) and fine aggregates. A fixed amount of SP was diluted with water separately and after dry mixing for around 1 min, this solution was added into the mix. The mixing continued for another 2 min until a mortar with uniform consistency was achieved. Additional SP was used (if needed) to maintain the same fluidity across all mixes. Fibers were thereafter added in two stages to ensure homogenous dispersion, aiming for a consistent flow table spread of 210–220 mm.

**Table 1:** Mix proportions used for the study.

Mix ID	*C/b	FA/b	SF/b	RS/b	SS/b	w/b	SP (kg/m^3^)	BF (^#^%Vol.)	PP (^#^%Vol.)
Control	0.35	0.60	0.05	0.40	0.00	0.24	04.87	2	0.11
HBF	0.35	0.60	0.05	0.40	0.00	0.24	06.56	1%12 + 1%18	0.11
BF1	0.35	0.60	0.05	0.40	0.00	0.24	03.50	1	0.11
SS30	0.35	0.60	0.05	0.28	0.12	0.24	08.75	2	0.11
SS100	0.35	0.60	0.05	0.00	0.40	0.24	14.22	2	0.11

*C/b—Cement to total binder ratio, ^#^%Vol.—% volume of total mix.

HBF-Hybrid length BF, BF1-1% Vol. of BF, SS30-30% replacement of RS with steel slag, SS100-100% replacement of RS with steel slag.

To evaluate the compressive strength of the composite, four cubes of side length 50 mm were cast for each mix at each combined temperature and fuel exposure level constituting a total of 120 cubes. Additionally, four cubes each were cast for control mix and SS100 for exposure to ETF alone which brings the total to 128 cubes. Also, for assessing the bending strength, three prisms of dimensions 350 × 40 × 40 mm were cast for each mix and for each combined temperature and fuel exposure level constituting a total of 90 prisms. The cubes and prisms were demoulded 36–48 h after casting and subjected to water curing for 28 days and then maintained at 25 ± 2 °C and 35 ± 2% relative humidity for another 28 days. This was done to ensure the specimen lose the excess moisture and the hydration process is almost complete.

### Test methods

**Figure 2:**
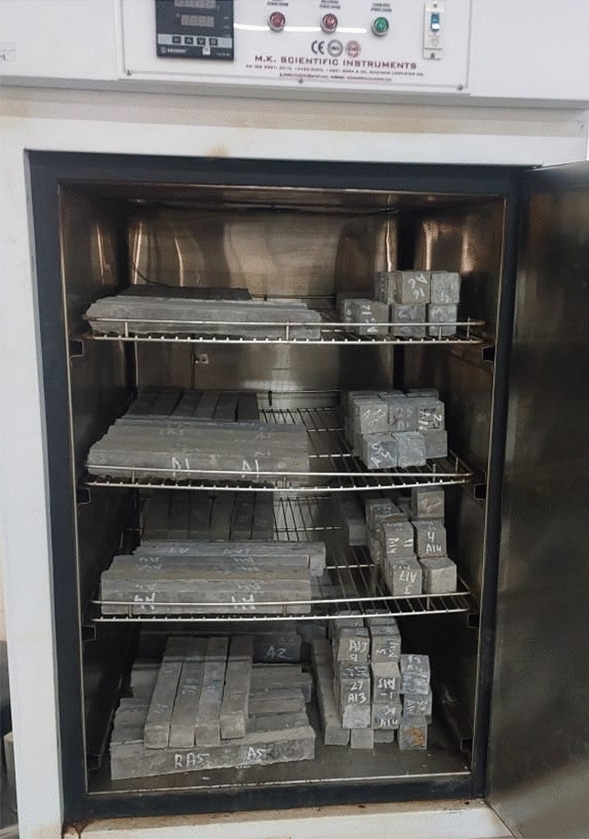
Environmental chamber with samples.

Following the 56-day curing period, the specimens were transferred into an environmental chamber for exposure to ETF and aircraft fluids as shown in Fig. 2.

**Figure 3:**
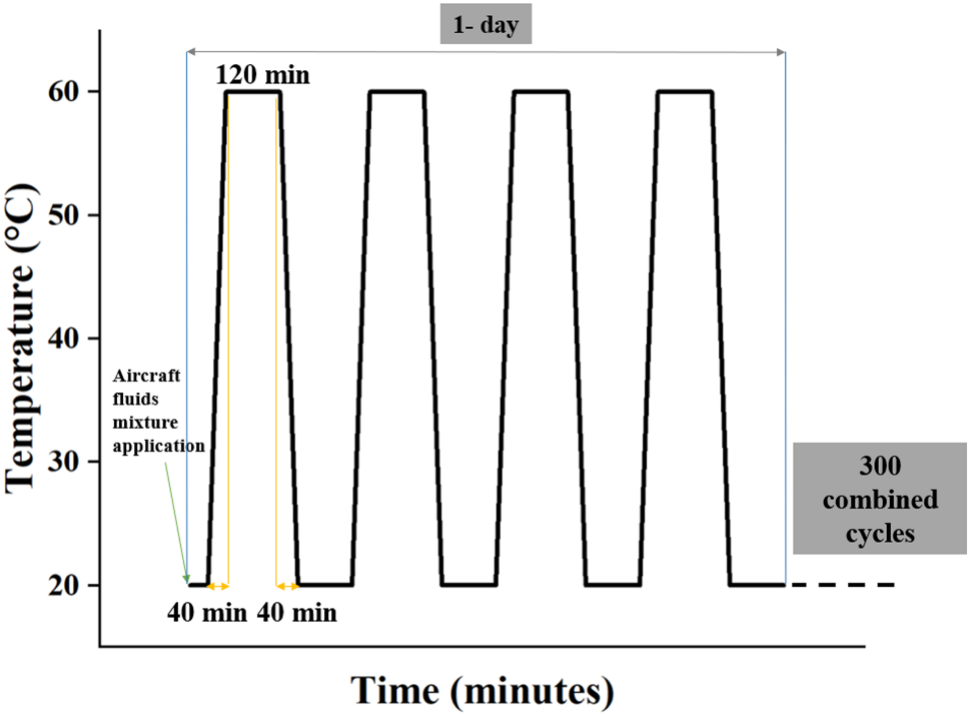
Temperature profile and aircraft fluid application schedule.

The aircraft fluids mentioned previously were mixed in equal parts and poured on the samples at the start of each day. Then the chamber programmatically cycled between 20 and 60 °C at a ramp rate of 1 °C per minute, including a hold period of two hours at each peak temperature. A 2-h hold period allowed the specimen's core to achieve the targeted temperature, ensuring the duration was enough for an isothermal state before initiating a thermal shock through cooling. Each cycle lasted approximately 5.5 h, allowing for 4 thermal cycles daily. The exposure conditions have been pictographically demonstrated in Fig. 3.

**Figure 4:**
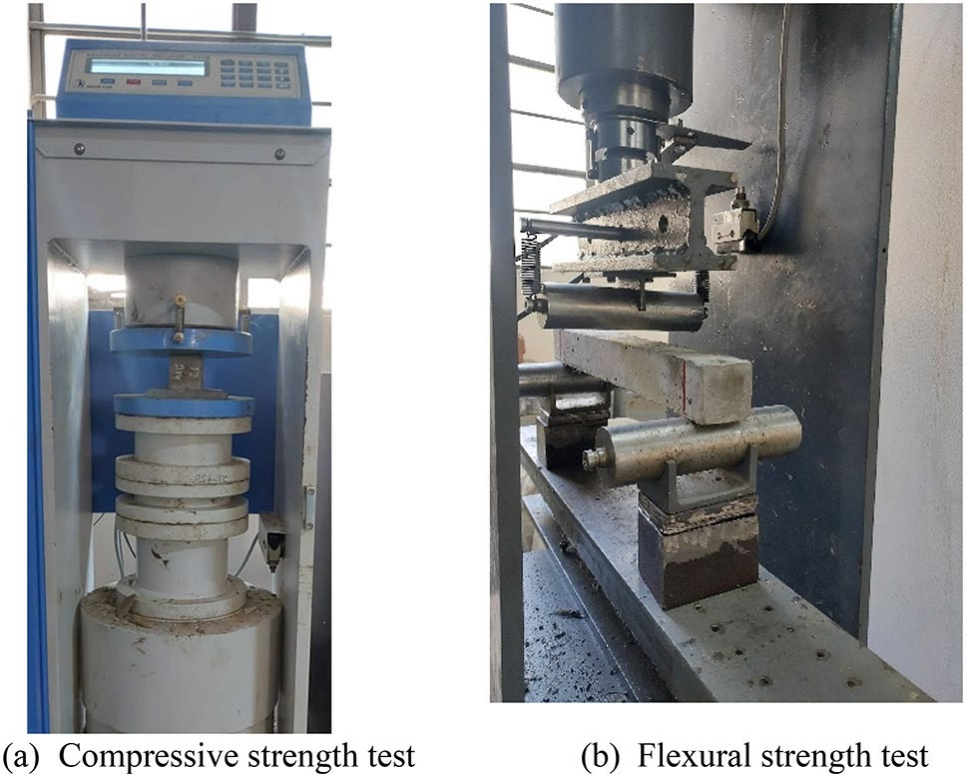
Testing of (**a**) Cube under compression (**b**) Prism under flexure.

The specimens were then removed at intervals corresponding to every 60 combined cycles (CCs) of ETF and aircraft fluid application, to assess their mechanical performance and durability under these conditions. The compressive strength tests were conducted on 50 mm side length cubes as per ASTM C39^26^. The surface of the cubes was polished using sandpaper as required and the cubes were tested in the direction perpendicular to the top surface during casting. Three-point bending tests were conducted on 350 × 40 × 40 mm prisms at a loading rate of 0.03 kN/s to assess the flexural strength of the cementitious composite. The length of the prisms was fixed to 350 mm with the nominal distance between the support as 300 mm as adopted by some recent literature based on ASTM C1609^27,28^.The load was applied at the mid-span of the prism. Rollers were used to support the two end to allow free horizontal movement. The test setup of both compressive and flexural strength test is further shown in Fig. 4. All the specimens were tested together to eliminate the effects of maturity at various stages of exposure.

To conduct SEM analysis, broken fragments of cubes were collected after compressive testing and stored in acetone for 72 h to arrest hydration reactions. The samples to be tested were coated with gold using a Quorum Tech sputter coater for 120–180 s before being tested for microstructure in Apreo S make field emission-scanning electron microscope. The same broken fragments were used for MIP analysis as well. MIP analysis was conducted using a Micromeritics AutoPore IV 9500 V1.09, with 0.10 to 60,000 psi pressure range. Mercury was introduced at a filling pressure of 0.50–0.51 psia with an equilibration time of 10 s. A portion of broken fragments were then powdered using ball mill and sieved through 75 µm sieve. DSC analysis was conducted on 50 ± 2 mg of this powdered sample (< 75 µm) in the temperature range of 0–800 °C at a heating rate of 5 °C/min in Argon environment.

## Results and discussion

### Surface characteristics

**Figure 5:**
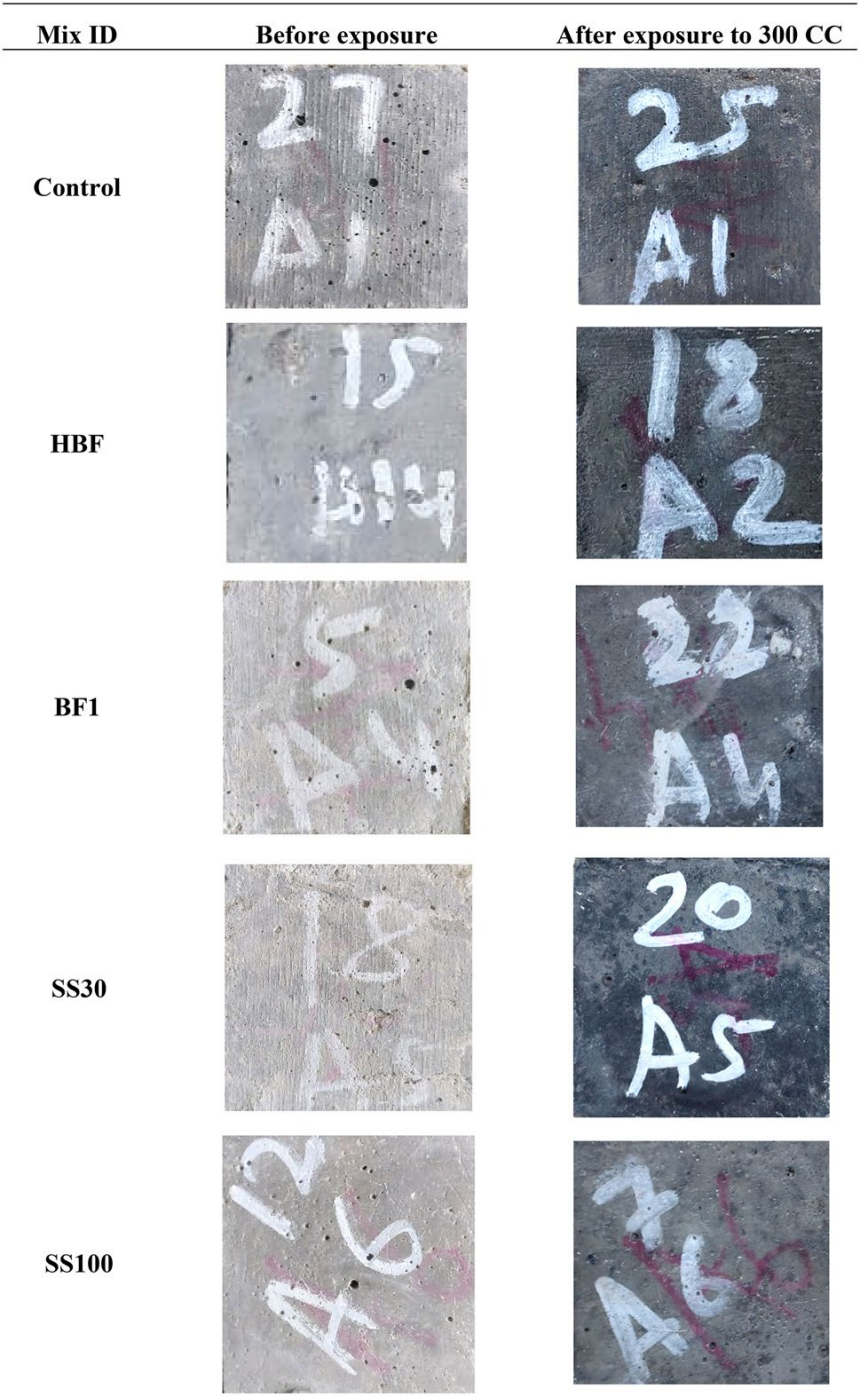
Surface characteristics of all the mixes before and after exposure to ETF indicating no signs of cracks.

Figure 5 demonstrates the variation in surface characteristics when exposed to 300 CCs as compared to the control specimens. The samples appeared darker in colour because of combined effect of aircraft fluids and heat.

Exposure to ETF can potentially cause cracking because of general drying and thermal expansion and contraction of various constituents inside concrete from variations in temperature. This is exacerbated when chemical attack from aircraft fluid is also present. Nevertheless, in the current study, incorporation of BF-PP possibly improved the tensile strength of the cementitious composite and controlled the occurrence of cracking^23,29^. In addition, at ETF temperatures, an enhancement of pozzolanic reaction of FA is observed^30,31^ which improved the strength of the composite and reduced cracking.

In terms of surface deterioration, Shill et al.^5,6,32^ suggested that exposure to aircraft fluids can remove aggregates from the surface of the concrete composite thus creating FOD on the airfield pavements. Therefore, in the current study, coarse aggregates were eliminated as they pose significant risk to the airline safety when detached from the pavement surface. Furthermore, no observations were made that indicated the loosening or removal of fine particles from the surface of the specimen even after 300 CCs. This is because of reduced availability of portlandite and calcite in the HVFA based system which are essential for reaction with aircraft fluids.

### Compressive strength observations

**Figure 6:**
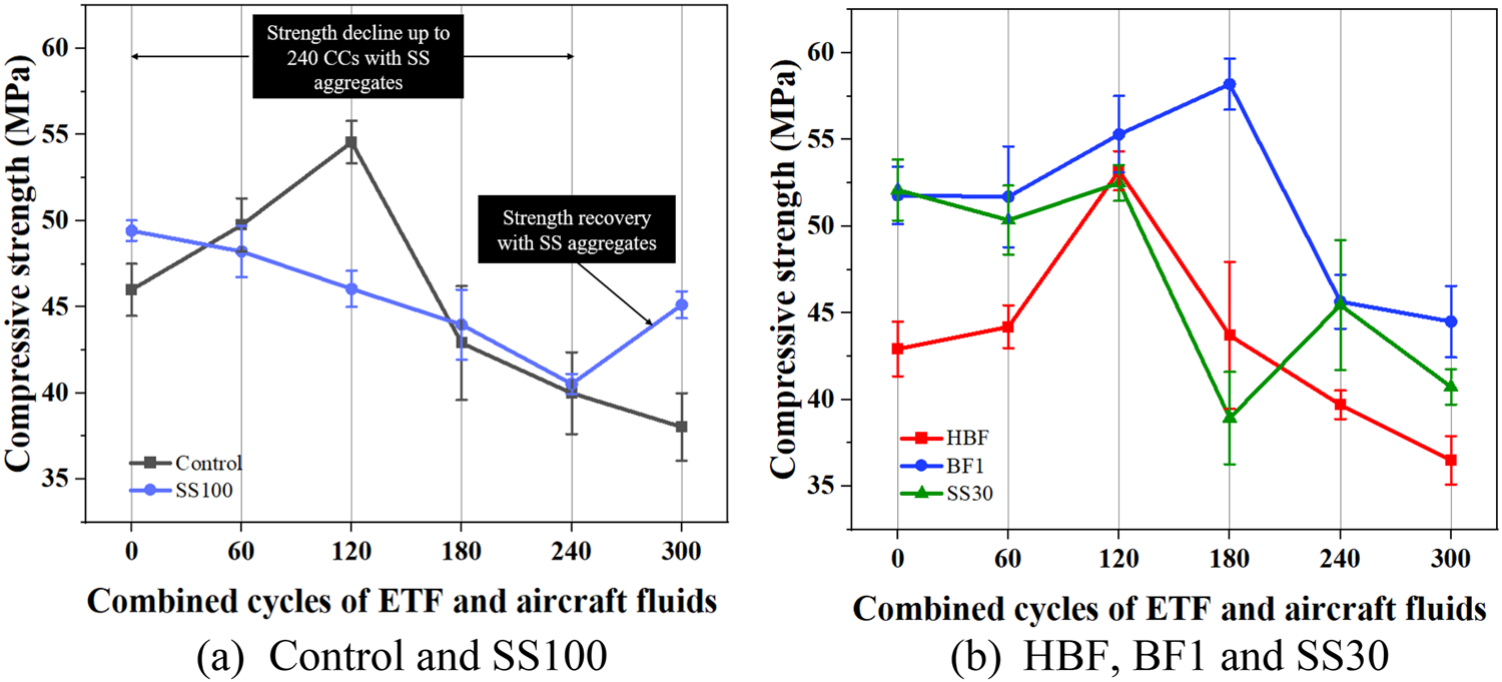
Compressive strength of all mixes with increasing combined ETF and aircraft fluid cycles.

Compressive strength of all the mixes with increasing CCs have been plotted as shown in Fig. 6. The control mix and HBF demonstrated similar compressive strengths of ~ 46 and ~ 43 MPa. However, decreasing BF volume by 1% in the mix BF1 led to a 12% increase in strength. Additionally, using SS aggregates (30% and 100% SS) improved compressive strength over the control mix.

Typically, the exposure to ETF has the potential to cause internal microcracking and damage to ITZ which leads to strength degradation. Additionally, the aircraft fluids cause saponification as discussed previously. Up to 120 CCs, the mixes containing only RS demonstrated an improvement in compressive strength owing to the improved pozzolanic and hydration reactions at higher ETF temperatures. This is coupled with delayed reaction of aircraft fluids in calcium deficient cementitious composites. However, upon exposure to 180 CCs, the effect of aircraft fluids became apparent. Significant decline in compressive strengths were observed in all mixes at this exposure level. This is attributed to the reaction of calcite with carboxylic acid to form calcium carboxylate salts, which reduces the aggregate matrix bond. After exposure to 180 CCs, a reduction in compressive strength (w.r.t 120 CCs) by 25% and 22% was observed for the control and HBF mixes, respectively. However, the mix BF1 demonstrated a continued improvement in strength till 180 CC. This mix contained lower fibre volume which may have reduced the porosity of the mix as also suggested in author’s previous work^19^. Consequently, a reduced depth of penetration of aircraft fluids resulted in a higher strength retention at this exposure level.

On the other hand, the mixes with steel slag aggregates, namely, SS30 and SS100, exhibited a reduction in compressive strength immediately after 60 CCs owing to the presence calcium-rich SS aggregates. These aggregates undergo accelerated hydration at ETF temperatures and release additional portlandite in the system that reacts with carboxylic acid from the aircraft fluids to form soap and water. This is in agreement with Arrhenius’s law which suggests that an increase in temperature increases the reaction rate^33^.

The formation of these soaps and salts reduces the elasticity and makes the cementitious composite weaker. The mix with 30% and 100% SS lost around 26% and 4% of its compressive strength (w.r.t 120 CCs) after exposure to 180 CCs.

**Figure 7:**
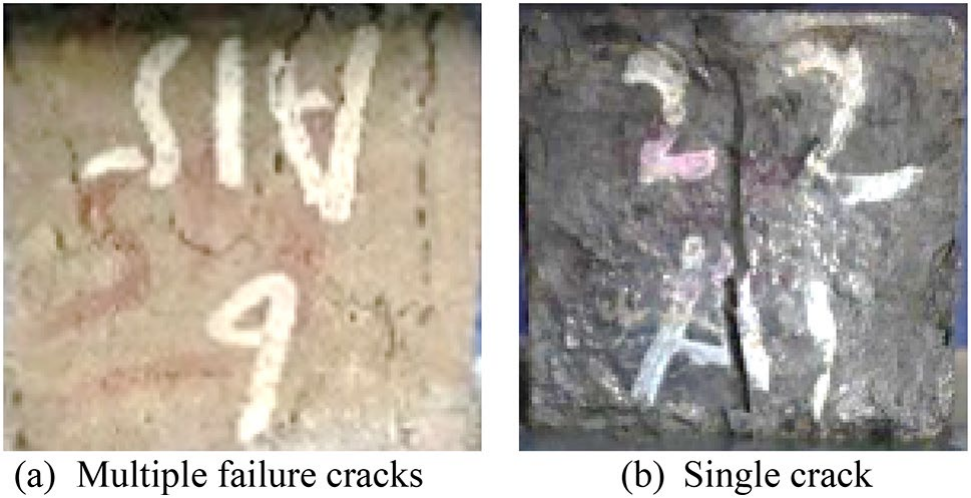
Comparison of failure pattern: (**a**) samples not exposed to CCs and (**b**) samples exposed to 180 CCs.

In terms of failure pattern, the mixes exposed to 180 CCs, demonstrated a brittle failure by a single crack. This is evident from the failure pattern observed during the testing of the cubical specimens under compression as shown in Fig. 7. The damage pattern in specimens not exposed to aircraft fluids exhibit failure by cracking through multiple failure plane.

After exposure to 240 and 300 CCs, strength degradation was less severe than at 180 CCs because of the lesser prevalence of portlandite and reactive calcium carbonate, which are crucial for the saponification. Interestingly, the mix SS100 demonstrated a minor improvement in compressive strength at 300 CCs. This may be because of the pozzolanic reaction of unreacted/partially reacted fly ash particles with the portlandite supplied from the SS aggregates from areas unaffected by the aircraft fluids. The degradation at this point is primarily through ETF. The increased porosity of the matrix through previous exposure to CCs also made the cementitious composite more resistant to ETF as observed through SEM images presented later in the paper. After exposure to 300 CCs, the loss in strength because of combined effect of ETF and aircraft fluids was approximately 17, 15, 14, 22, and 9% in control, HBF, BF1, SS30 and SS100 specimens, respectively. As can be observed, the performance of FRCC with 100% SS aggregates is better at both 0 CCs (or at ambient temperature) and after exposure to 300 CCs which makes them a suitable candidate to replace RS.

### Flexural strength

**Figure 8:**
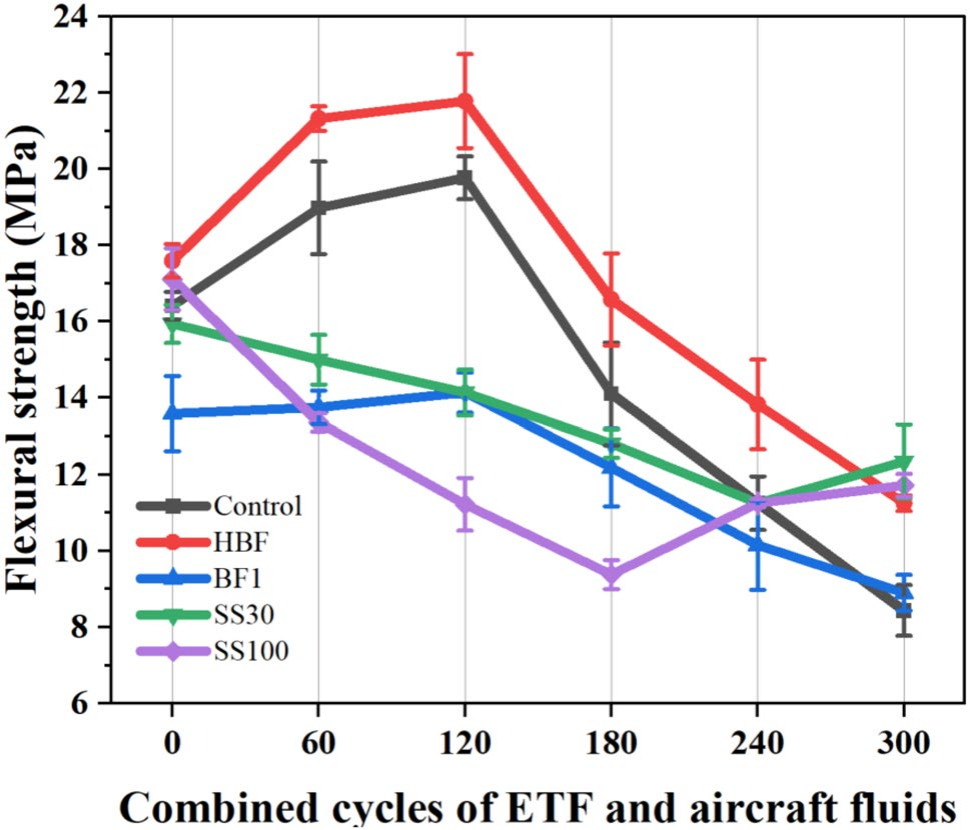
Flexural strength variations of all mixes up to 300 CCs.

Figure 8 provides the flexural strength of all the mixes up to 300 CCs. As can be observed, hybrid length basalt fibres increased the flexural strength by ~ 7% because of better bridging effects. On the other hand, reducing BF volume by 1% in the mix BF1 decreased flexural strength by ~ 19%. Replacement of RS with 30% and 100% SS aggregates did not significantly impact the bending strength.

The mixes with only RS demonstrated an improvement in flexural strength up to 120 CCs owing to the elevated temperature accelerated reaction of FA. The control mix and HBF gained strength ~ 15% and 21%, respectively over the flexural strengths achieved by the specimens at 0 CCs. The mix BF1 demonstrated a marginal increase in flexural strength. Upon exposing the control, HBF and BF1 mixes to 120 CCs, smaller increment in strengths of approximately 5, 3 and 3% respectively were observed.

After 180 CCs when sample is sufficiently saturated with aircraft fluids in areas with portlandite and calcite, deterioration was observed. All the mixes with RS demonstrated a flexural strength decline between 6 and 15% after 180 CCs with respect to 0 CCs. After exposure to 240 CCs, a further decline of 22–32% was observed in these mixes. The loss exhibited by the control, HBF, and 1BF was reported to be ~ 32, 22, and 25%, respectively. The mix HBF performed better than the control mix because of better anchorage of longer BF, while the mix BF1 performed better because of its lower porosity that reduces the ingress of deleterious aircraft fluids. After exposure to 300 CCs, the control mix lost ~ 49% of its flexural strength while the strength loss of HBF and BF1 was observed to be ~ 36% and ~ 35%, respectively.

The mixes SS30 and SS100 both demonstrated a decline in flexural strength immediately after exposure to the first 60 CCs as opposed to an increase in flexural strength as observed in mixes with RS. This is because of the chemical attack from the aviation fluids on the calcium-rich matrix causing internal saponification. The decline in flexural strength of SS30 was found to be gradual whereas the mix SS100 lost nearly 46% by 180 CCs. The strength decline for SS30 is continued till 240 CCs and then a reversal is observed at 300 CCs where a part of the flexural strength is recovered. This mix retained the maximum relative residual flexural strength of ~ 77% after exposure to 300 CCs. The mix SS100 demonstrated a strength redemption from 240 CCs and finally exhibited a higher relative residual flexural strength greater than that of the control mix.

Complete replacement of RS with SS aggregates may not be suggested for airfield pavements subjected to aviation fluids as significant early-stage strength loss is observed. On the other hand, a 30% replacement of RS with SS aggregates can be considered, as it brings positive effects of SS aggregates such as strong ITZ and possible participation in pozzolanic reaction in areas unaffected by aircraft fluids.

### SEM analysis

#### General microstructural observations

**Figure 9:**
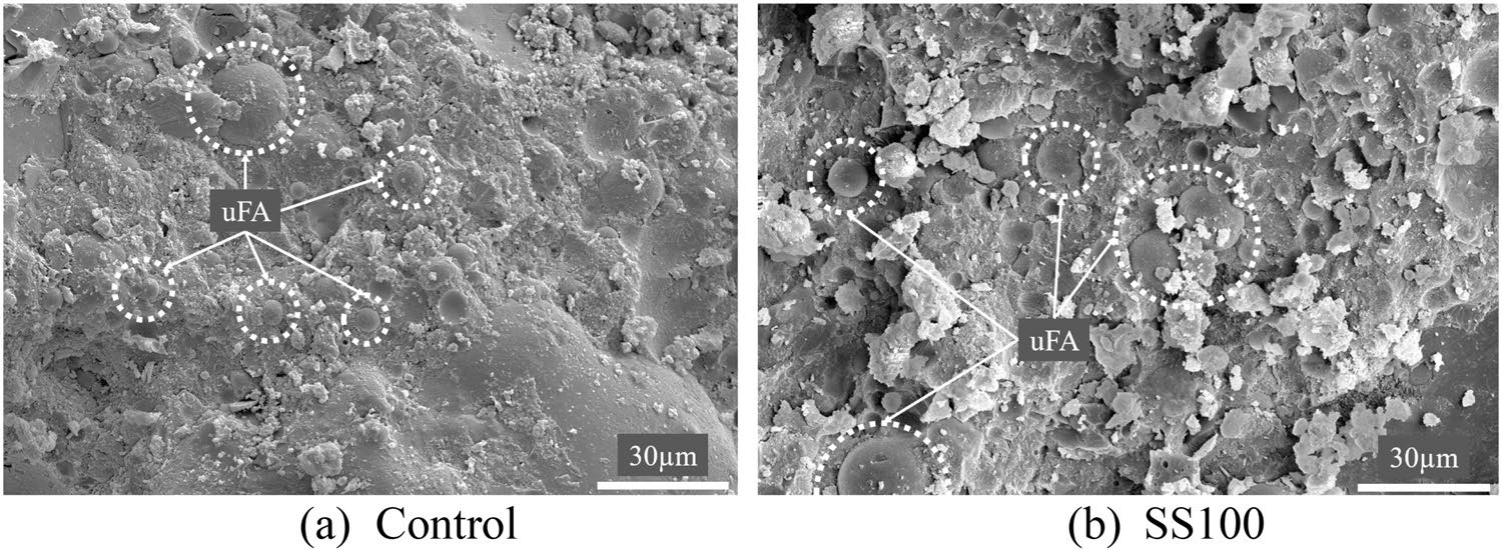
Microstructure demonstrating abundant unreacted FA content in (**a**) Control and (**b**) SS100.

Figure 9 presents the microstructure of the control mix and SS100 for comparison. These two mixes are chosen given that their behaviour can be used to determine the underlying mechanisms of variations observed in the study. Both the mixes demonstrate abundant presence of unreacted FA (uFA) particles.

**Figure 10:**
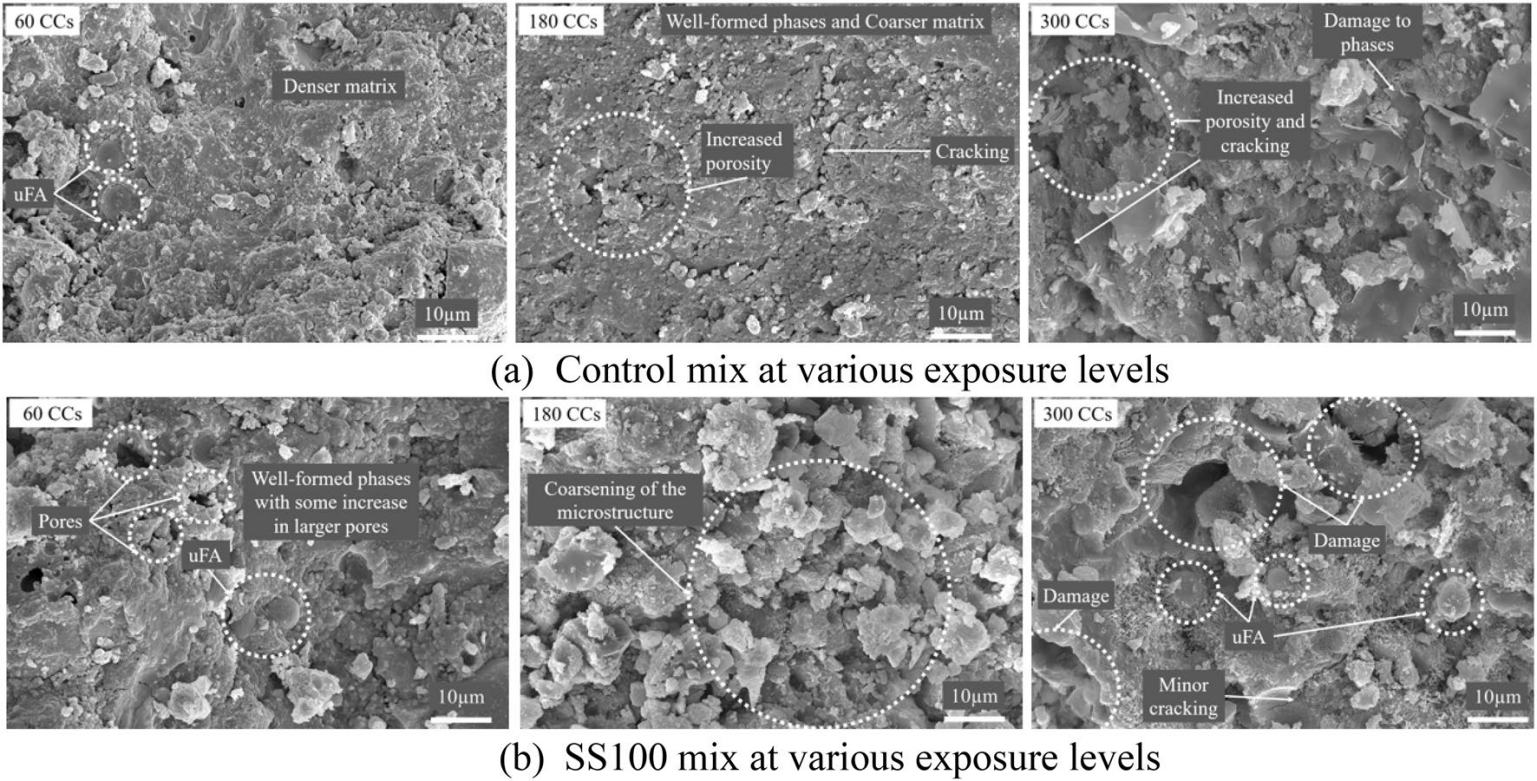
Microstructure of (**a**) Control and (**b**) SS100 at various exposure levels.

Figure 10 presents the microstructural variations of the control and SS100 when exposed to combined effect of aircraft fluids and ETF. After exposure to 60 CCs, the microstructure of control mix demonstrated an improvement in density while the microstructure of SS100 remained similar to the sample which was not exposed to the combined cycles. Consequently, the strength of the control mix increased by ~ 8% and the strength of SS100 remained approximately the same.

**Figure 11:**
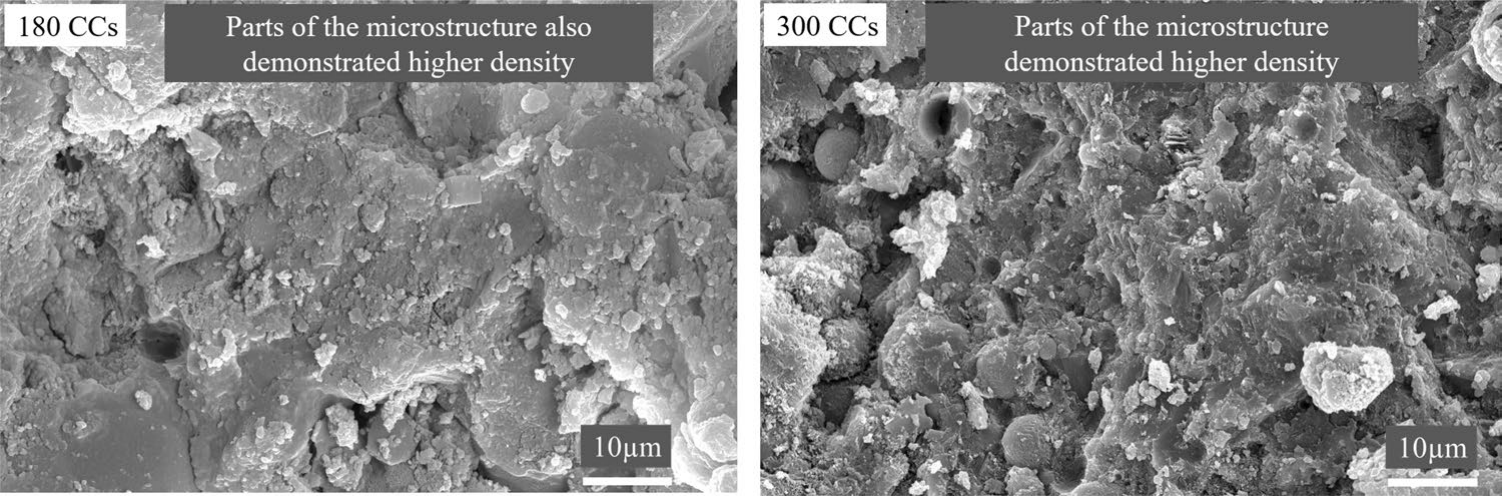
Parts of microstructure with higher density in SS100 without exposure to aircraft fluids.

After exposure to 180 CCs, the microstructure of the control mix began to demonstrate signs of damage with coarsening of the microstructure and increased porosity distributed unevenly throughout the microstructure as shown in Fig. 10. This delay in initiation of damage to the cementitious composite is because of positive effect of HVFA utilization. The unreacted FA particles initially densified the matrix upon exposure to ETF and delayed the percolation of aircraft fluids. Usually, ETF alone causes degradation of microstructure by cracking and pore coarsening which was delayed by the incorporation of TPE in the form of HVFA. For the mix SS100, severe coarsening was observed in most regions and others demonstrated a formation of denser microstructure as demonstrated in Figs. 10b and 11, respectively.

After 300 CCs, the control mix exhibited a damaged microstructure with an increase in porous areas and cracking in addition to the clear signs of damage to the hydration phases. On the other hand, SS100 exhibited significantly enhanced density in comparison to that observed in case of the samples exposed to 180 CCs. This may be because of continued pozzolanic reaction from the parts not vulnerable to aircraft fluids. The presence of SS aggregates continually supplies the portlandite required for continued pozzolanic reaction which enhances the strength of the composite.

#### ITZ observations

**Figure 12:**
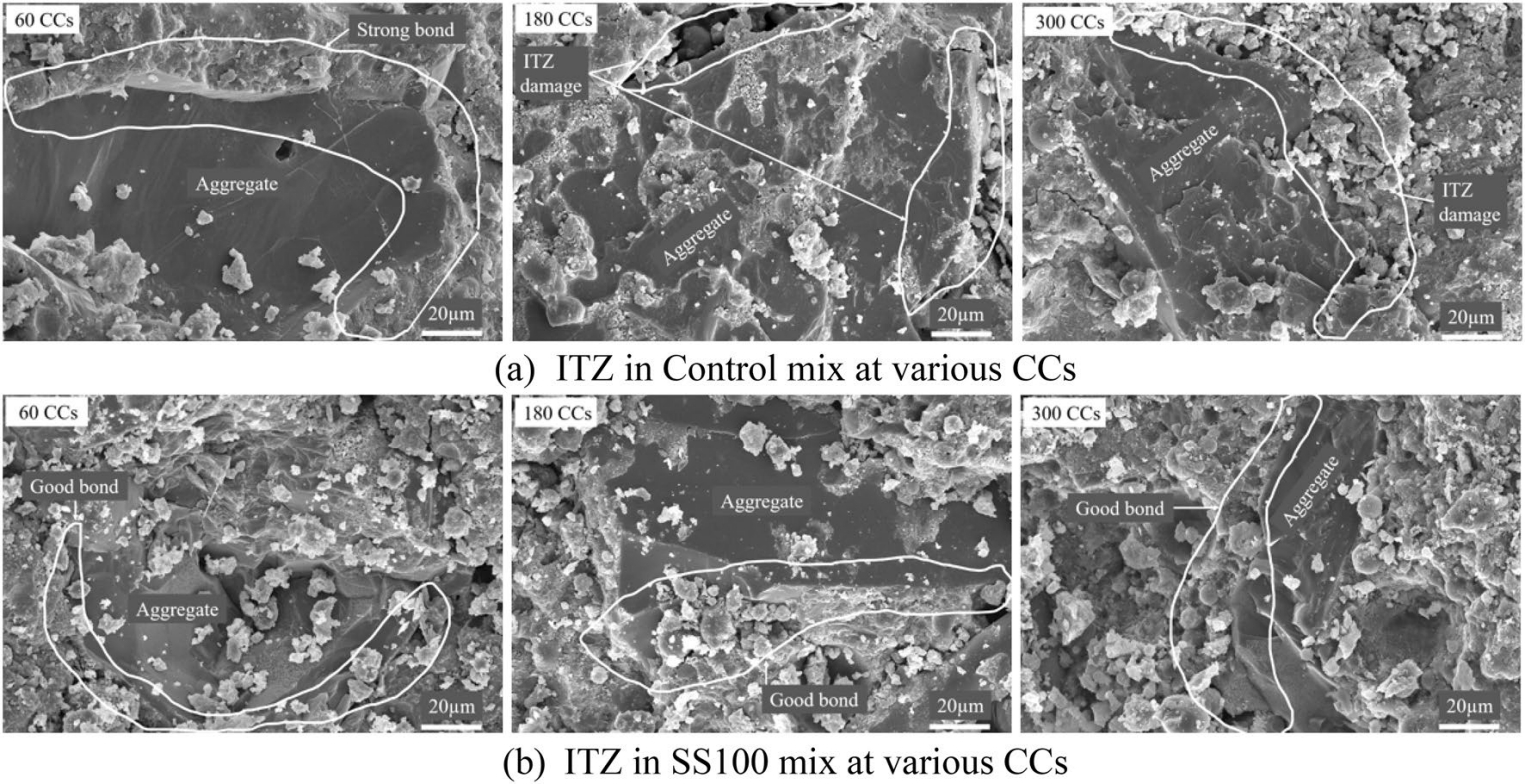
Variation of ITZ observed in (**a**) Control mix and (**b**) SS100 at 60, 180, and 300 CCs.

The combined action of ETF and aircraft fluids have the potential to reduce the bonding between aggregate and paste phase. Therefore, a special focus on variation of ITZ behaviour with increasing combined cycles is essential and a comparison between RS and SS fine aggregates has been made in Fig. 12.

At 60 CCs, the ITZ of aggregates in control mix demonstrated a strong bond owing to accelerated pozzolanic reaction of uFA at higher temperatures of ETF. At 180 CCs, the effect of ETF (causing differential thermal expansion) and aircraft fluids was apparent and the bond between aggregate and cement paste deteriorated. Consequently, this weakening of the ITZ contributed to a steep reduction in compressive strength as observed earlier. At 300 CCs, similar poor bond was observed between aggregate and paste phase.

On the contrary, the bond between SS aggregate and paste phase remained unaffected throughout. The strong interlocking of SS aggregates is because of its angular shape and rough texture in addition to the formation of hydration products on its surface when exposed to higher temperatures. Furthermore, the differential thermal expansion between SS aggregates and the paste phase is lesser provided they have similar chemical composition.

### MIP analysis

**Figure 13:**
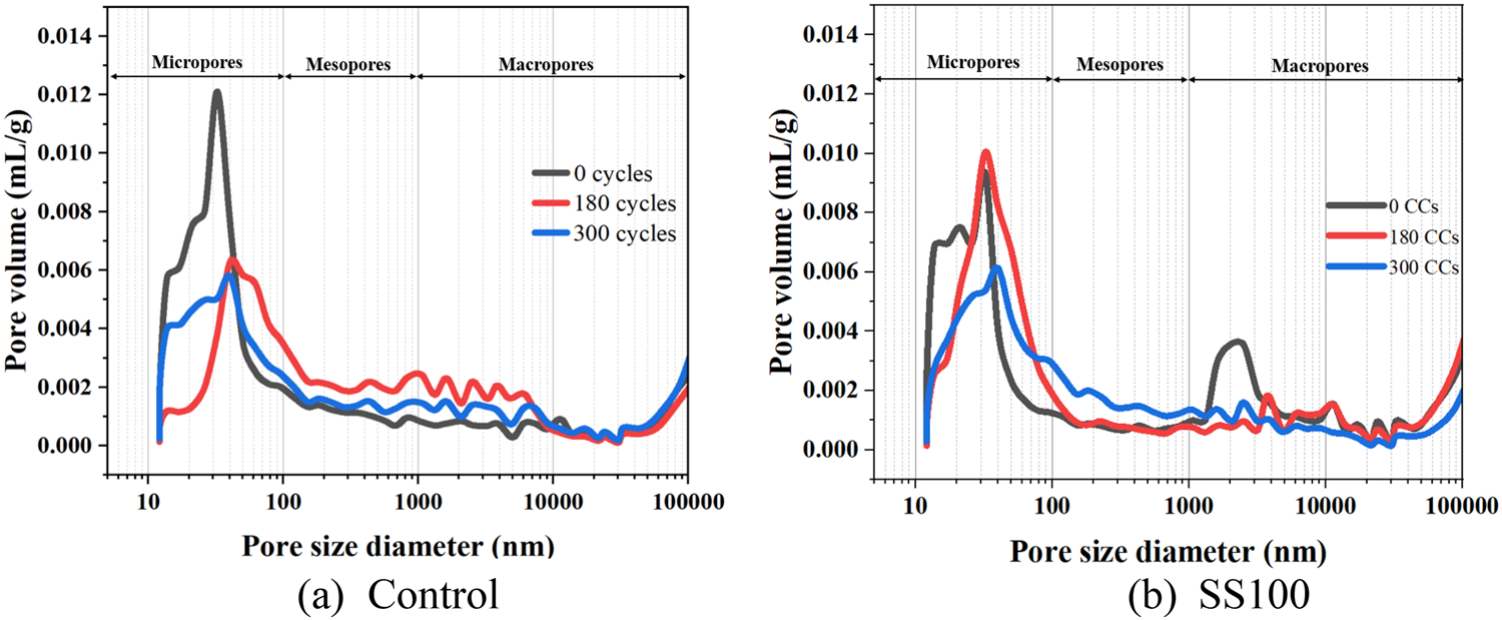
MIP analysis of (**a**) Control and (**b**) SS100 after exposure to 0, 180 and 300 CCs.

**Figure 14:**
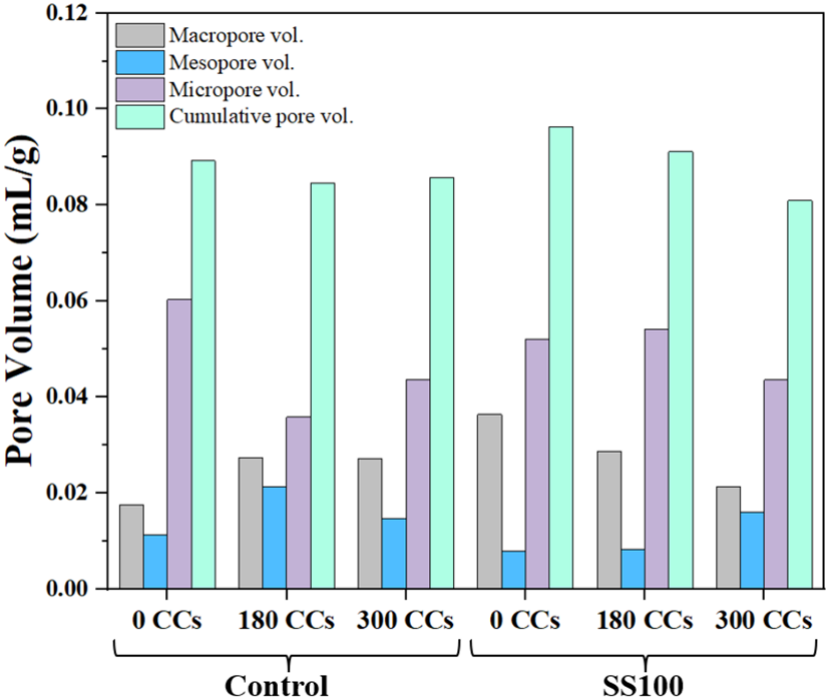
Pore volume information of the control and SS100 mix after exposure to various CCs.

Significant variation in porosity was observed through SEM analysis because of exposure to CCs and therefore, MIP analysis was utilized to gain further insights into the porosity of the material. Figure 13 provides the information on pore size distribution of control and SS100 after exposure to various combined cycles. The same information has been quantified in Fig. 14.

The effect of ETF on reduction of total pore volume is apparent in control mix from Figs. 13 and 14. After exposure to 180 CCs, significant reduction in the early part of the micropore region is observed. Despite the reduction in smaller sized pores in micropore region, saponification-induced elasticity reduction can cause a decline in compressive strength of the composite. Also, pore coarsening can be observed in the micropore region from a visible shift of the curve towards right towards pores of higher diameter. Also, the curve for pore volume in the mesopore and macropore regions shifted up representing an increase in larger pores. This is supported by an increase in the volume of mesopores and macropores as observed from Fig. 14. These larger pores upon application of compressive load may have developed a failure plane that caused a brittle failure as shown in Fig. 7. Consequently, a steep decline in compressive strength was observed despite reductions in total pore volume. After exposure to 300 CCs, an increase in micropores was observed with respect to 180 CCs. The macropores remained the same as that of 180 CCs while a reduction in mesopores were observed. Overall, exposure to 300 CCs caused an increase in the total pore volume with respect to samples exposed to 180 CCs.

For SS100, the application of 180 CCs caused an increase in the pore volume in micropore and mesopore region. This behaviour is contrary to the control mix where a significant reduction in micropores was observed. This was also observed through SEM where severe coarsening of microstructure was observed as demonstrated in Fig. 10b. This caused a steep decline in compressive strength at 180 CCs. Interestingly, upon continual exposure to ETF and aircraft fluids up to 300 CCs, a significant reduction in total volume of micropores and macropores was observed. This is consistent with compressive strength observations and was possibly due to the improvement caused by pozzolanic reaction of FA aided by the portlandite supplied by SS aggregates in regions not exposed to aircraft fluids.

MIP analysis was further used to understand the performance of mix BF1 (1% BF + 0.11% PP), which demonstrated better compressive strength in comparison to the control mix (2% BF + 0.11% PP). Upon MIP analysis of the BF1 samples, cumulative pore intrusion volume of 0.0877, 0.0776 and 0.0796 mL/g was observed when exposed to 0, 180 and 300 CCs respectively. These values were found to be lower than the control and SS100 (Fig. 14) which indicates lower porosity and hence, may have resulted in the better performance of the BF1 mix.

### DSC analysis

**Figure 15:**
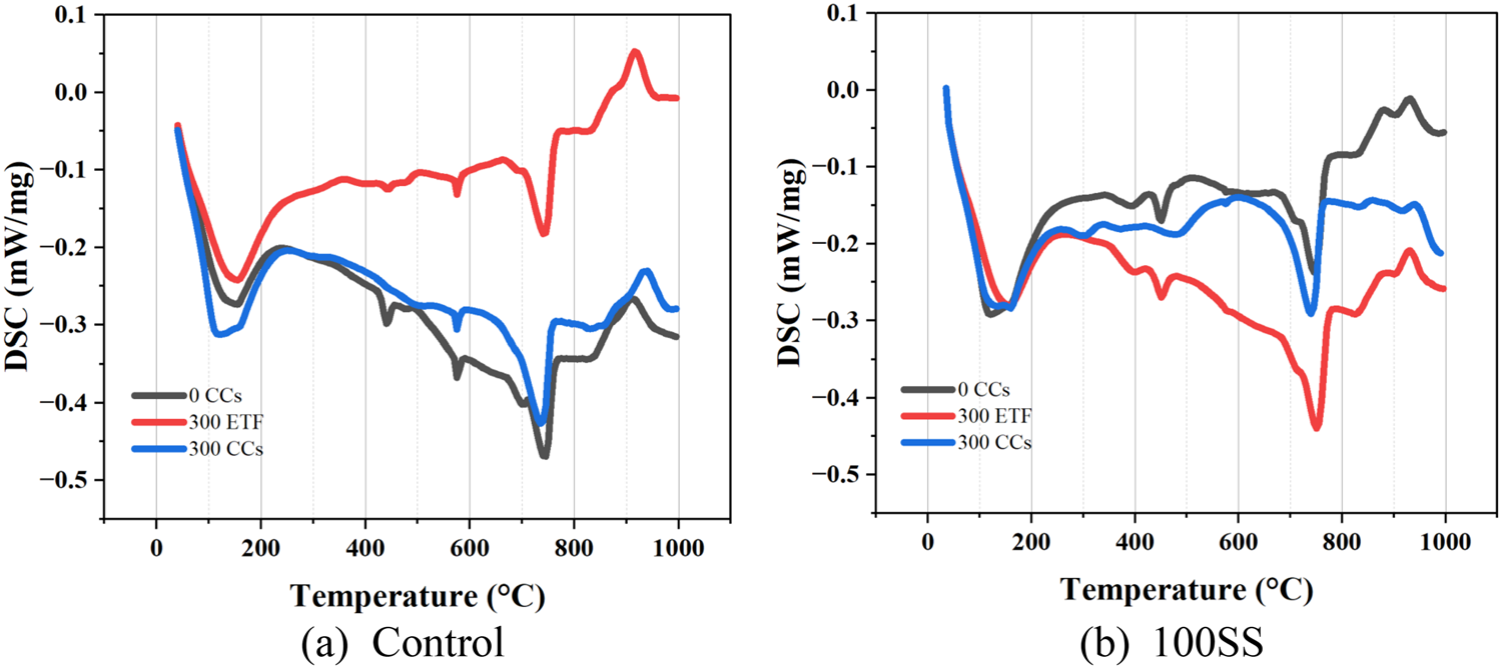
DSC curves for (**a**) Control and (**b**) SS100 after exposure to 0 CCs, 300 CCs and 300 ETF.

Figure 15 provides DSC curves for control mix and SS100 after exposure to various exposure conditions. The application of ETF and aircraft fluids causes significant changes to the composition of the FRCC. In all the samples, endothermic peaks were observed near 120–150 °C, 450 °C, and 750 °C. These peaks signify the dehydration of free and physically bound water, dehydroxylation of portlandite, and decarbonation of calcium carbonate, respectively^34,35^.

In case of control mix, the endothermic peak corresponding to portlandite decomposition was absent after exposure to 300 CCs. The removal of portlandite from the system was because of accelerated pozzolanic reaction and saponification. Also, water is a by-product of saponification reaction as discussed earlier. Consequently, the peaks corresponding to dehydration exhibited an increase in energy indicating occurrence of saponification. On the other hand, clear reduction in energy for dehydration is observed in the sample subjected to 300 ETF. The consumption of calcium carbonate by esters of fatty acid to form calcium salts is evident from the reduction in endothermic peak at 300 CCs.

For SS100, it can be observed that portlandite is removed after exposure to 300 CCs. However, the portlandite existed in samples exposed to 300 ETF alone which is mainly contributed by SS aggregates (as this endothermic peak is missing from the control mix exposed to ETF). This portlandite provided by SS aggregates (from regions unaffected by aircraft fluids) improved the pore structure of the cementitious composite as demonstrated using MIP. A higher endothermic peak associated with decarboxylation was observed in case of SS100 subjected to 300 ETF. The calcium carbonate was consumed by the formation of calcium salts when exposed to combined ETF and aircraft fluids. The endothermic peak corresponding to dehydration was similar to unexposed specimen. This may be because of the utilization of water in pozzolanic and hydration reactions.

## Discussion on the damage mechanism of composite under ETF and aircraft fluids

The percolation of aviation fluids, the concentration of calcium compounds (namely hydroxide and carbonates), and the exposure temperature critically influence the deterioration of airfield pavements.

The current study demonstrated that systems with reduced concentrations of calcium hydroxide and calcium carbonates are less susceptible to soap formation, thus are more apt for airfield pavement construction. When HVFA is utilized as TPE with RS in FRCC, the detrimental effects of aircraft fluids are not immediately evident. This is attributed to the consumption of accessible portlandite in the pozzolanic reaction of FA as demonstrated through DSC analysis. Consequently, an improvement in strength was observed up to 120 CCs suggesting formation of a denser microstructure. Additionally, a reduction in portlandite content may decrease the coefficient of thermal expansion of the system, thereby reducing uncoordinated deformation within the cementitious composite, which delays microcracking^36,37^. Contrarily, an immediate decline in compressive and flexural strengths was observed for mixes containing calcium rich SS aggregates suggesting their vulnerability.

The ETF, typically detrimental to cementitious composites, has been observed to beneficially delay the penetration of deleterious fluids for the adopted material system. However, once sufficient aircraft fluids infiltrate the sample because of repeated application and encounter regions containing calcium carbonate and/or calcium hydroxide, a process of saponification ensues, rendering the FRCC brittle and deteriorating the bond between the aggregate and the paste phase. As observed through MIP analysis, despite reduction in the total pore volume, saponification-induced decrease in elasticity can lead to lower compressive strength in the composite. This can also be aided by an increase in larger sized pores which could form a failure plane under compressive stresses.

Furthermore, surface cracking caused by general drying, and expansion–contraction was not observed. Also, severe internal cracking was avoided. This is attributed to incorporation of BF-PP, which potentially enhanced the tensile strength of the cementitious composite and protected the matrix from cracking. Also, the positive effect of BF content is clear considering significantly higher flexural strengths were noted for control and HBF mixes in comparison to BF1, especially after exposure to CCs. Conversely, it adversely impacts the compressive performance of the cementitious composite. Therefore, BF volume must account for optimal performance in terms of both compressive and flexural strengths.

## Conclusion

This study focussed on the combined effects of ETF and aircraft fluids on the HVFA based cementitious composite. Macro properties in terms of compressive and flexural strengths were investigated at various exposure levels up to 300 CCs and the damage mechanism was uncovered using SEM, DSC, and MIP. The following conclusions can be derived based on the findings of this study.

- Compressive strength of the mixes with RS, initially increased up to 120 CCs. An increase of ~ 18, ~ 23 and ~ 6% was observed in the control mix, HBF and BF1 respectively. A steep decline in compressive strength was observed after exposure to 180 CCs. Compressive strength continued to decline at a slower rate for 240 and 300 CCs.
- The mixes with SS aggregates demonstrated immediate decline in compressive strength after exposure to 60 CCs. This was because of the calcium-rich SS aggregates that release portlandite at ETF temperatures. The microstructure coarsened significantly after exposure to 180 CCs. A higher strength was observed when exposed to 300 CCs proving the positive effect of utilization of SS aggregates.
- HVFA as TPE effectively delays the loss in flexural strength up to 120 CCs. Both fibre hybridization and fibre volume reduction were found to be effective strategies for reducing the extent of flexural strength deterioration. Utilization of SS aggregates reduces the flexural strength initially, which is then redeemed with increasing CCs.
- The microstructure of the FRCC displayed varied characteristics, with some regions exhibiting higher density and others showing porous structures, suggesting that the combined exposure to ETF and aircraft fluids leads to diverse effects.
- MIP analysis further revealed the impact of incorporation of HVFA. The pore volume of the control mix in the micropore region reduced upon exposure to 180 CCs and 300 CCs, however, an increase in larger sized pores in mesopore and macropore region was observed. Contrarily, in case of SS100, a steep rise in pore volume in the initial micropore range was observed. Interestingly, after exposure to 300 CCs, a decline in pore volume of micropore and macropore range was observed. This indicates that the SS aggregates which were not affected by aircraft fluids supplied portlandite for continued pozzolanic and hydration reactions. At the same time, pore volume in mesopore region increased.

Overall, the HVFA-based cementitious composites are less vulnerable to attacks from aircraft fluids considering the carboxylic acid, phosphoric acid, and esters of fatty acids all require calcium hydroxide and calcium carbonate for soap formation. Upon exposure to ETF, the reactivity of FA particles is enhanced and pozzolanic reaction starts to deplete the available portlandite. Therefore, the HVFA-system can delay and reduce the extent of saponification. A complete replacement of RS with SS aggregates is not recommended given the rapid decline in both compressive and flexural strengths. A lower replacement level of 30% should be considered which yields better results in comparison.

## Data Availability

Data will be made available on request from the corresponding author.
